# Comparison of the Donor Age-Dependent and *In Vitro* Culture-Dependent Mesenchymal Stem Cell Aging in Rat Model

**DOI:** 10.1155/2021/6665358

**Published:** 2021-05-14

**Authors:** Katarzyna Siennicka, Aleksandra Zołocińska, Tomasz Dębski, Zygmunt Pojda

**Affiliations:** Department of Regenerative Medicine, Maria Sklodowska-Curie National Research Institute of Oncology, Warsaw, Poland

## Abstract

Clinical experiments suggest that mesenchymal stem cells (MSCs) may be useful for tissue repair therapies or treatment of the autoimmune disorders. There is still lack of consensus concerning the age limit of MSC donors, majority of researchers suggest the autologous MSC therapies of patients not exceeding age limit of 55-60 yrs. The purpose of our study was to compare the selected parameters of MSCs from adipose tissue (adipose stem cell, ASC) collected from young and old rats of ages corresponding to patient's ages 25 yrs. and 80 yrs., respectively. The differences of parameters of ASCs from young and old animals were compared with the differences between ASCs from short-term (3 passage) and long-term (30 passage) *in vitro* culture. Cell morphology, surface marker expression, growth potential, metabolic activity, *β*-galactosidase activity, clonogenic potential, angiogenic potential, and differentiation ability of ASCs from young and aged animals and from *in vitro* cultures at 3rd and 30th passages were compared and analyzed. It may be concluded that ASCs may be applied for autologous transplantations in aged patients. Comparison of ASC aging dynamics depending on host aging or *in vitro* culture duration suggests that long-term *in vitro* culture may affect ASCs more than natural aging process of their host. We suggest that ASCs expanded *in vitro* prior to their clinical use must be carefully screened for the possible aging effects resulting not only from donor age, but from the duration of their *in vitro* culture.

## 1. Introduction

The ClinicalTrials.gov database search (2020) results in 1071 clinical experiments with the use of mesenchymal stem (stromal) cells (MSCs) for treatment of the wide variety of injuries and diseases. Out of this number, 391 trials include autologous MSC treatment. There is no consensus on the age limit for the MSC donors. Several reports confirm MSC aging (*β*-galactosidase activity, telomere length, and telomerase activity) related to host age, but the data on the therapeutical usefulness of MSCs from the senile donors are insufficient [1–6]. Even more limited is the information concerning the relationship between *in vitro* aging caused by the long-term *in vitro* MSC expansion and their morphological and functional parameters which are customary analyzed in the process of the qualification of MSCs for therapeutic applications.

We used the rat model, corresponding to the human ASC (adipose stem cell, subclass of mesenchymal stem cell) donors of the age of 25 yrs. and 80 yrs. for the comparison of the cell parameters commonly attributed to age (*β*-galactosidase activity) and parameters important for therapeutic usefulness of ASC for the clinical applications (proliferative potential, metabolic activity, differentiation into adipo-, chondro-, and osteogenic lineages, and the capacity to induce angiogenesis [7–9]. For the comparison of ASC “aging” resulting from the increase of donor age and “aging” resulting from the *in vitro* culture duration, we compared ASCs, collected from young (6 mth) and aged (2 yrs.) rats from short-term (3 passage) *in vitro* culture and long-term (30 passage) *in vitro* culture.

## 2. Materials and Methods

### 2.1. Collection and Isolation of ASCs from Rat Adipose Tissue

Male Wistar Albino Glaxo inbred rats aged 6 mth (309.8 ± 21.6 g) and 2 yrs. (353.5 ± 19.1 g) were used throughout experiment. Inhalation method (isoflurane overdose) was used for euthanasia of animals. White adipose tissue was collected from inguinal, perigonadal, and perirenal regions. Pooled adipose tissue from different regions of each animal was digested in 0.075% solution of collagenase (from Clostridium histolyticum, Sigma-Aldrich, USA) in phosphate-buffered saline (PBS, Life Technologies Co, USA) for 30 min in 37°C. Resulting suspension was mixed with 10% FBS (Fetal Bovine Serum, Life Technologies Co, USA), filtered and washed with PBS. Cell numbers and viability were tested using ADAM-MC automated cell counter (NanoEnTek Inc., USA), cells were suspended in DMEM medium (Life Technologies, USA) plus 10% FBS and used for further experiments. All animal experiments were approved by the II Local Bioethical Committee in Warsaw (Approval number: 71/215/30/06/2015) and performed according to the Guidelines for the Regulation of Animal Experiments.

### 2.2. In Vitro Culture of Rat ASCs

Cells were cultured in 75 cm^2^ plastic flasks (Nunclon, USA), 5 × 10^5^ cells per flask, 37°C, 5% CO_2_, and 95% humidity. After achieving 80% confluence, cultures were trypsinized (0.25% trypsin, Life Technologies, USA, 37°C), resuspended in DMEM +10% FBS, and transferred to the new culture flask.

### 2.3. Evaluation of MSC Morphology (Cell Shape, Granulation, Surface Markers)

Cell morphology was evaluated using Juli™ Stage Real-Time History Recorder (NanoEnTek Inc., USA), and cell dimensions and shape were compared. Cell granulation and expression of surface markers were analyzed using flow cytometer (FACS Calibur and program CellQUEST pro, Becton Dickinson, USA). Expression of CD29, CD11b, CD90, CD45, and CD34 surface antigens (markers) was quantitatively evaluated, and cell granulation was estimated using forward scatter (FSC) and side scatter (SSC) parameters.

### 2.4. Doubling Time and Growth Rate

ASCs were seeded on multiwell plates in proportion of 1 × 10^4^ cells per 9.6 cm^2^ flat well surface. Cultures were performed in the chamber with controlled gas composition (5% CO_2_-enriched atmosphere, 95% humidity) (CMV1500 Baker Russkin Co, USA), equipped with the microscope system enabling constant picture monitoring (Juli™ Stage NanoEnTek Inc., USA) using program Real-Time History Recorder. The performed determinations allowed for microscopic observations, taking photos, and counting cells always in the same area, at different time points. Observations and photographic recordings of the marked areas were carried out every 5 hours until the cells were fully confluent. From the designated and registered areas, cells were counted from the moment of their adhesion to the plastic until the end of the experiment, taking into account the recording time. Growth rate and doubling time were calculated using free program Doubling Time Online Calculator (http://www.doubling-time.com/compute_more.php).

### 2.5. Metabolic Activity

Metabolic (mitochondrial) activity of ASCs was measured using commercially available MTS test kit Cell Titet 96® Aqueous One Solution Cell Proliferation Assay (Promega, USA). Product of the reduction of 3-(4,5-dimethylthiazol-2-yl)-2,5-diphenyltetrazolium bromide to water-soluble formazan was measured using spectrophotometer Multiscan GO (Thermo Fisher Scientific, USA). Quantity of formazan product as measured by the amount of 490 nm absorbance is directly proportional to the number of living cells in culture. 5 × 10^4^ cells per well were seeded on 24-well plates and cultured in DMEM supplemented by 10% FBS. Metabolic activity was tested in 3 time points: after 24 h, 7 days, and 21 days of culture.

### 2.6. Clonogenic Potential

Cell clonogenic potential (CFU-F test) was performed in 6-well culture plates (2 ml DMEM +10% FBS, 2.6 × 10^2^ ASC per well, 5% CO_2_, 37°C, 95% humidity, and 7 d culture). Cell colonies (>50 cells/colony) were scored under inverted microscope following May-Grunwald/Giemsa staining.

### 2.7. ASC Differentiation (Adipo-, Osteo-, and Chondrogenesis)

Plastic-adherent ASCs were stimulated into adipo-, osteo-, and chondrogenesis differentiation by the use of commercially available differentiating media (LONZA, Switzerland), according to producer's protocols (adipogenic induction medium and adipogenic maintenance medium for adipogenesis, osteogenic induction medium for osteogenesis, and complete chondrogenic induction medium for induction of chondrogenic differentiation). Effects of adipogenic differentiation were evaluated under microscope following staining of cultures with Oil Red (Sigma-Aldrich, USA) and using real-time PCR for CEBPA (Rn00560963_s1), ADIPOQ (Rn00595250_m1), and PPAR*γ* (Rn00440945_m1) gene expression specific for adipogenesis. Osteogenic differentiation was evaluated after cell staining with Alizarin Red S (Sigma-Aldrich, USA) and using real-time PCR for osteogenesis-specific genes ALPL (Rn01516028_m1), RUNX2 (Rn01512298_m1), and IBSP (Rn00561414_m1). Chondrogenic differentiation was evaluated under microscope after Masson Trichrome staining and by real-time PCR for chondrogenesis-specific genes COMP (Rn00563255_m1), SOX9 (Rn01751069_mH), and ACAN (Rn00573424_m1). Gene expression was determined using real-time PCR (Roche LightCycler®96, RFN). The reaction mixture (20 *μ*l) contained 9 *μ*l of reverse transcription cDNA (concentration 100-150 *μ*l of suspended in nuclease-free water (Sigma-Aldrich, USA)), 10 *μ*l of TaqMan Universal PCR Master Mix, NoAmpErase UNG (2x), and 1 *μ*l of TaqMan probe. The reaction steps were as follows: denaturation step—1 cycle: 50°C for 120 s and 95°C for 600 s followed by 40 cycles of elongation step: 95°C for 15 s and 60°C for 60s, and cooling step at 37°C. GAPDH (Rn01775763_g1) and ACTB (Rn00667869_m1) were used as reference genes in each sample.

### 2.8. Angiogenic Potential and Paracrine Proangiogenic Effect of ASC

#### 2.8.1. Angiogenic Potential of ASCs

Angiogenic potential of ASC was evaluated by the application of 2 independent tests: *tubule formation assay* and *sprouting from 3D spheroids assay.* Tubule formation assay was performed using 96-well plates covered with Matrigel® (Corning®, USA) with 1 × 10^4^ cells per well added and suspended in 100 *μ*l of EGM-2 (LONZA, Switzerland), incubated for 20 h (37°C, 5% CO_2_, and 95% humidity). Test results were evaluated by identification of the formation of tubules under inverted microscope. 3D sprouting assay was performed in the round-bottom 96-well plates. Spheroid formation was observed after 24 h incubation of 750 cells per well in 1 : 4 mixture of methylcellulose (R&D Systems, USA) and EGM-2 medium.

#### 2.8.2. Stimulatory (Paracrine) Angiogenic Effect of ASCs on HUVEC Cells

Stimulatory effects of conditioned media from ASC on the HUVEC cell cultures were tested. Positive control cultures were performed in 6-well HUVEC cell cultures in Matrigel in EGM-2 medium plus 0.5% FBS, and negative control cultures were done similarly but in EGM-2 medium plus 0.5% FBS added. HUVEC cells were incubated for 20 h (37°C, 5% CO_2_, and 95% humidity). Conditioned media were collected from ASC (young or senile rats, 3 passage or 30th passage) cultures (6-well plates, 3 × 10^5^ cells per well, Matrigel with 2 ml EGM − 2 + 0.5% FBS medium per well, 37°C, 5% CO_2_, and 20 h incubation) and added into HUVEC cultures (6-well plates, Matrigel plus conditioned medium, 37°C, 5% CO_2_, 95% humidity, and 20 h). The criterium of the positive paracrine effect of ESC on HUVEC cells was tubule formation in HUVEC cultures observed under visible light contrast phase inverted microscope (Olympus CKX41, Japan).

### 2.9. *β*-Galactosidase (SA-*β*-Gal) Activity

ASCs (2 × 10^4^ cells/well) were cultured for 24 h in 24-well plates (37°C, 5% CO_2_, and 95% humidity) and tested for *β*-galactosidase activity with Senescence *β*-Galactosidase Staining Kit (Cell Signaling Technology) according to producer's manual. Cell ratio of blue-stained versus nonstained cells was scored under Nikon Eclipse Ti microscope (Japan) using the computer program Nis-Elements AR Analysis 4.00.12.

### 2.10. Statistical Analysis

Statistical analysis was performed using Statistica 13 PL computer program. After getting the results with Shapiro-Wilk test, data were analyzed with one- or two-factor ANOVA variance tests, ANOVA repeated-measures analysis of variance, linear regression, Levene test for variancy homogeneity, and post hoc Tukey HSD and NIR tests. Comparison of means of specific group with controls was done using Dunnett test. Statistically significant differences were analyzed at *p* < 0.05 level (graphic descriptions: statistical significance levels described as ^∗^*p* < 0.05, ^∗∗^*p* < 0.01, and ^∗∗∗^*p* < 0.001; M: statistical mean; SE: standard error).

## 3. Results

### 3.1. ASC Morphology

**Figure 1:**
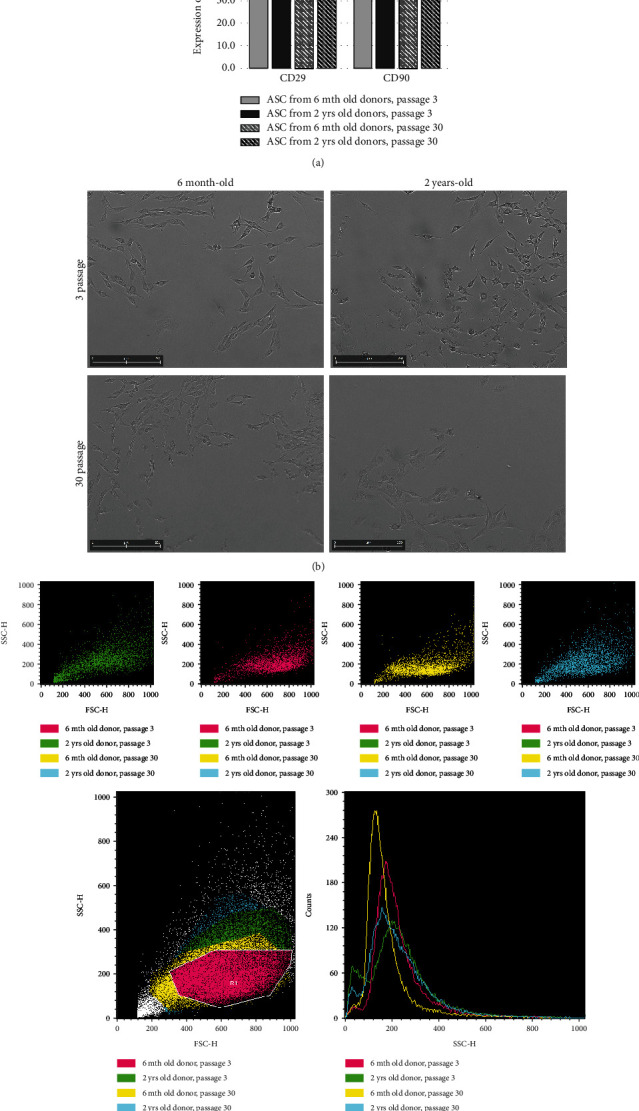
(a) Comparison of the cell surface expression CD29 and CD90 from short-term (passage 3) and long-term (passage 30) *in vitro* cultures of rats 6 mth old and 2 yrs. old; (b) morphology of ASCs from short term (3 passage) of 6 mth old donors and 2 yrs. old donors and of ASCs from long-term (30 passage) culture of 6 mth old donors and 2 yrs. old donors; (c) flow cytometry analysis (FSC/SSC) of the granulation of ASCs from 2 yrs. donors, 3 p. (passage) and 30 p. and 6 mth donors, 3 p. and 30 p. Comparison of the extent of granules is shown below.

We did not observe any significant differences between CD29 and CD90 surface marker expression between young (6 mth) and old (2 yrs.) donors and between cells from short-term (3 passage) and long-term (30 passage) *in vitro* cultures (Figure 1(a)). The transition of cell shape from fibroblast-like to epithelial-like was typical for the *in vitro* aging of ASC cultures, and the pattern of this phenomenon was similar for ASCs derived both from young and old donors (Figure 1(b)). Adversely, the granularity of ASCs increased with the donor age, but was independent from the *in vitro* culture duration (Figure 1(c)).

### 3.2. Functional Characteristics of ASC from Young and Old Donors, Cultured for Short or Long Time In Vitro

Analysis of the growth rate and doubling time of ASCs from young (6 mth) or old (2 yrs.) rats cultured *in vitro* for short time (3 passage) or long time (30 passage) did not show any significant differences (Table 1). The practical aspect of this observation is that the expansion of ASCs in bioreactors, being standard for most clinical protocols may be performed similarly for cells obtained both from young and old donors.

**Figure 2:**
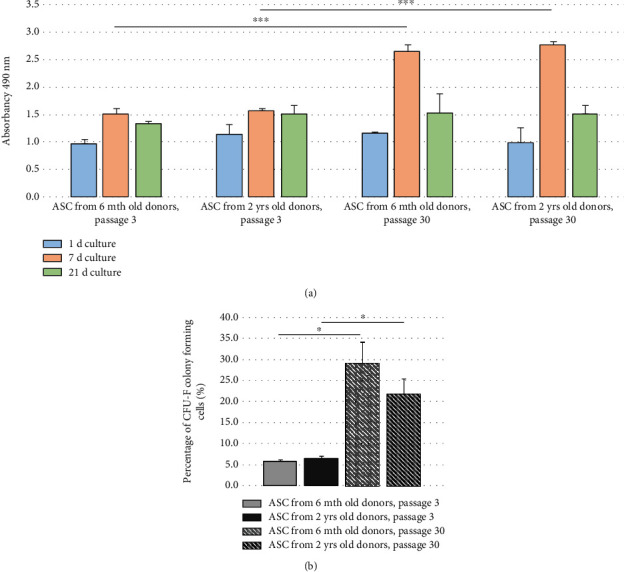
(a) ASC metabolic activity from passage 3 (p. 3) and passage 30 (p. 30) from donors aged 6 mth and 2 yrs., respectively. 1, 7, or 21 d culture means the duration of MTS assay. ^∗∗∗^*p* < 0.001; (b) clonogenic potential (CFU-F test) of ASCs from 6 mth and 2 yrs. donors, short-term (3 p.) and long-term (30 p.) *in vitro* culture. ^∗^*p* < 0.05.

Results of MTS test comparing metabolic activity of ASCs (7 d cell preincubation time) have shown significant increase (*p* < 0.001) of the activity of ASCs' long-term cultured for 30 passages over the activity of cells from short-time culture (3 passages) (Figure 2(a)). The increase of ASC activity related to their *in vitro* culture time may be important when considering their therapeutic effects. Since this observation is new and results are rather unexpected, further research is needed for estimation of its clinical importance.

Clonogenic potential, measured by CFU-F test, was not influenced by the donor age. There was, however, significant (*p* < 0.05) difference between CFU-F percentage in short-term and long-term cultures of ASCs, both from young and old donors (Figure 2(b)). The finding is rather unexpected and needing further studies, and it may be concluded that the duration of *in vitro* culture prefers proliferation of cells having greater colony-forming potential and probably being at earlier stage of cell maturation.

### 3.3. Differentiation Potential of ASCs from Young or Old Donors, Cultured for Short or Long Time In Vitro

**Figure 3:**
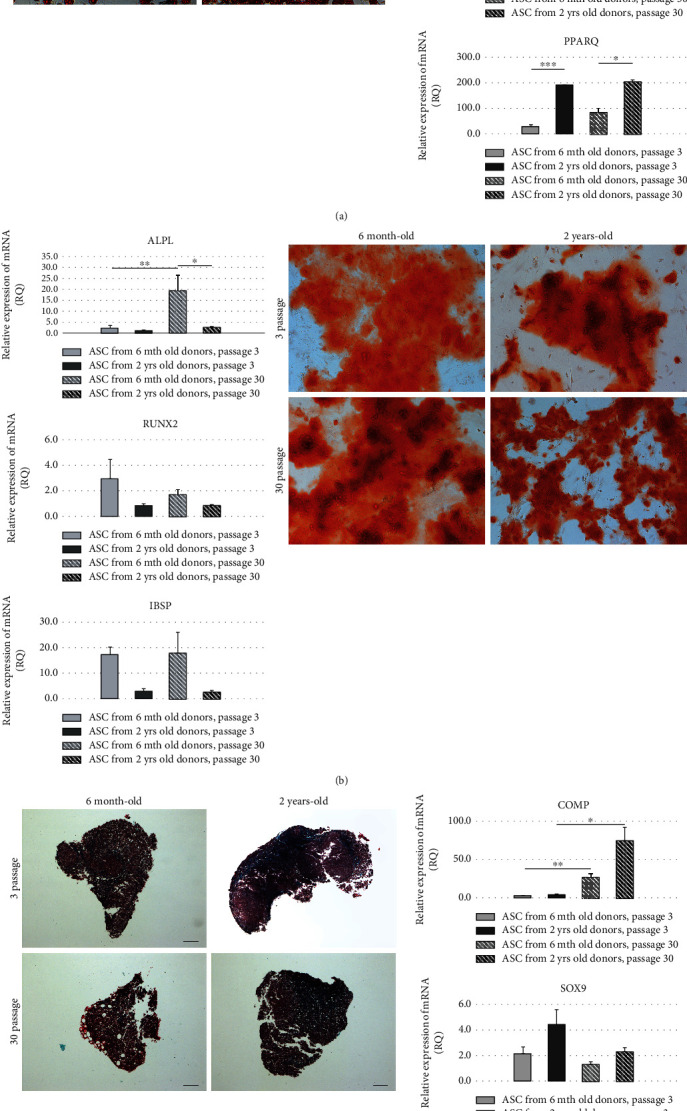
(a) Adipogenic differentiation of ASCs from 6 mth donors, p. 3; 2 yrs. donors, p. 3; 6 mth donors, p. 30; 2 yrs. donors, p. 30 and comparison of adipogenesis-specific gene expressions of CEBPA, ADIPOQ, and PPAR*γ* in adipogenic-stimulated ASCs from young (6 mth) and aged rats (2 yrs.) cultured for 3 or 30 passages; (b) osteogenic differentiation of ASCs from 6 mth donors, p. 3; 2 yrs. donors, p. 3; 6 mth donors, p. 30; 2 yrs. donors, p. 30 and comparison of osteogenesis-specific gene expressions of ALPL, RUNX2, and IBSP in adipogenic-stimulated ASCs from young (6 mth) and aged rats (2 yrs.) cultured for 3 or 30 passages; (c) chondrogenic differentiation of ASCs from 6 mth donors, p. 3; 2 yrs. donors, p. 3; 6 mth donors, p. 30; 2 yrs. donors, p. 30 and comparison of chondrogenesis-specific gene expressions of COMP, SOX9, and ACAN in adipogenic-stimulated ASCs from young (6 mth) and aged rats (2 yrs.) cultured for 3 or 30 passages.

Adipogenic differentiation of ASCs has manifested by the change of cell shape (from elongated to more rotund) and the presence of oil red lipid vesicles inside cells. Real-time PCR (CEBPA, ADIPOQ, and PPAR*γ*) revealed higher adipogenic potential of ASCs from old donors (*p* < 0.01) and increased adipogenic potential of ASCs in long-term cultured ASCs (*p* < 0.01) (Figure 3(a)).

Osteogenic potential of ASCs was tested by Alizarin Red staining. Higher expression of osteogenesis-related genes (ALPL, RUNX2, and IBSP) was observed in ASC cultures from young donors (confirmed by real-time PCR), *p* < 0.05 (Figure 3(b)). *In vitro* culture time did not influence the osteogenic potential of ASCs, both from young and old donors.

Chondrogenic differentiation was tested by histology staining of collagen fibers and real-time PCR for genes COMP, SOX9, and ACAN (Figure 3(c)). Higher gene expression was observed for old donors (*p* < 0.001 for ACAN gene). Long-term *in vitro* culture increased expression of COMP (*p* < 0.01 in young donors and *p* < 0.05 in old donors) when compared to the corresponding data from short-term *in vitro* culture. There was no visible difference in the intensity of Masson trichrome staining of samples from young or old donors or short-term or long-term *in vitro* cultures (Figure 3(c)).

### 3.4. Proangiogenic Potential of ASCs from Young or Old Donors or from Short-Term or Long-Term In Vitro Cultures

**Figure 4:**
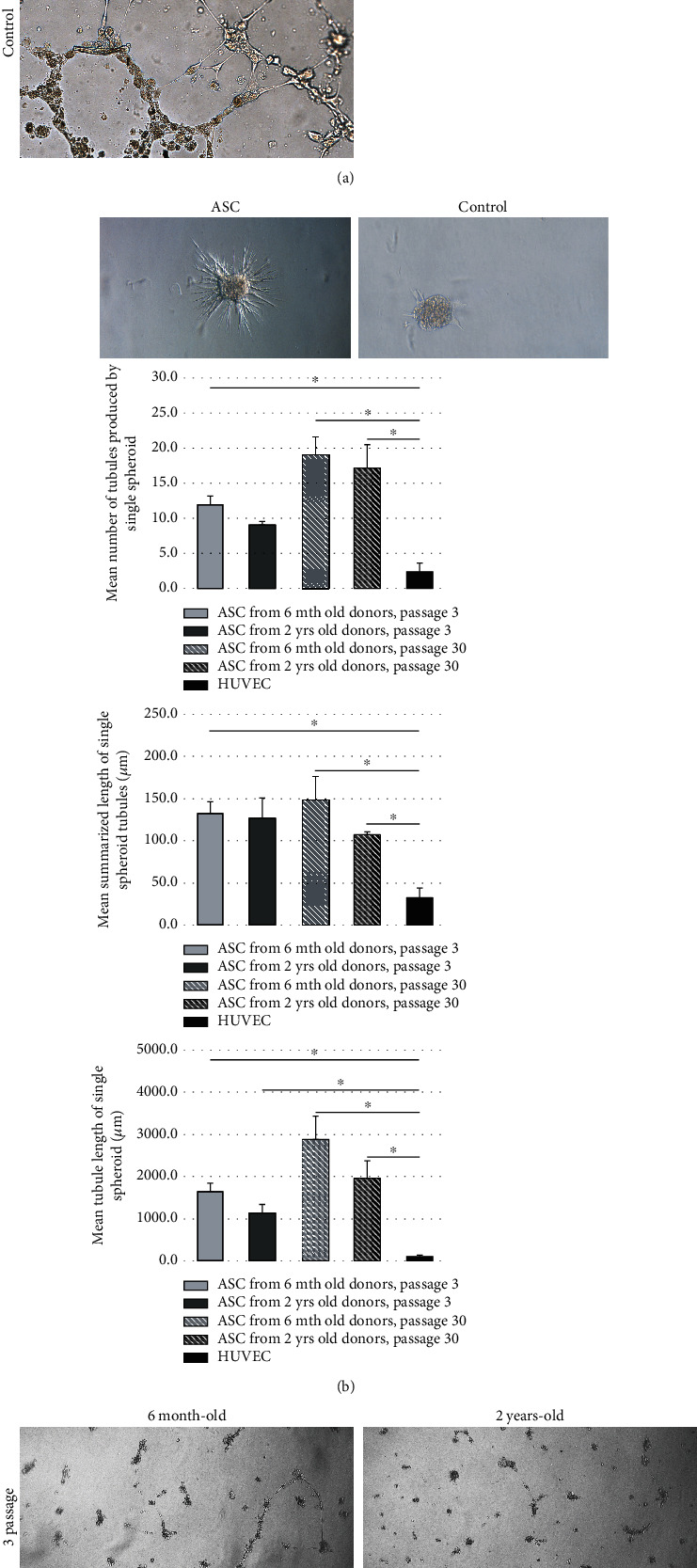
(a) Tubule formation by Matrigel-cultured ASCs from 6 mth rats, p. 3; 2 yrs. rats, p. 3; 6 mth rats, p. 30; 2 yrs. p. 30 and HUVEC cell culture as a control; (b) tubule-like structures emerging from spheroids cultured in collagen gel: ASC and HUVEC (control); mean numbers of the tubules produced by spheroids composed by ASCs from 6 mth and 2 yrs. donors, short-term (3 p.) and long-term (30 p.) in vitro culture. HUVEC spheroids served as controls. ^∗^*p* < 0.05; total length of tubules per spheroids composed by ASCs from 6 mth and 2 yrs. donors, short-term (3 p.) and long-term (30 p.) *in vitro* culture. HUVEC spheroids served as controls. ^∗^*p* < 0.05; mean tubule length produced by spheroids composed by ASCs from 6 mth and 2 yrs. donors, short-term (3 p.) and long-term (30 p.) *in vitro* culture. HUVEC spheroids served as controls. ^∗^*p* < 0.05; (c) HUVEC-produced tubules in Matrigel stimulated by supernatants from cultures of ASCs from 6 mth donors, p. 3; 2 yrs. donors, p. 3; 6 mth donors, p. 30; 2 yrs. donors, p. 30. Negative controls were HUVEC cultures in EGM − 2 + 0.5% FBS; positive controls were HUVEC cultures in EGM − 2 + 20% FBS.

ASCs from young donors and from long-term *in vitro* cultures exhibited higher potential for tubule formation when compared to old donors and to short-term *in vitro* cultures (Figure 4(a)). Number of tubules produced by spheroids formed by ASCs (Figure 4(b)) were higher in long-term cultures both from young and old donors, and all ASC-formed spheroids produced more tubules than HUVEC control cultures (*p* < 0.05, Figure 4(b)).

Summarized length of tubules produced by single spheroid was higher for young versus old donors and in both groups higher for long-term versus short-term cultures and higher in ASC-formed spheroids than HUVEC-formed spheroids (*p* < 0.05).

Mean tubule lengths were higher in spheroids formed by ASCs from young donors, higher in spheroids formed by ASCs from long-term cultures, and all types of ASC-formed spheroid forming tubules are significantly longer than HUVEC controls (*p* < 0.05).

Control HUVEC cultures in Matrigel (0.5% FBS) did not produce tubules, and addition of ASC-conditioned media resulted in tubule production; no statistically significant differences between the effects of conditioned media from ASCs from young or old donors and short-term or long-term cultures were observed (Figure 4(c)).

When summarizing the angiogenesis test results, it may be concluded that both ASCs per se and ASC culture-conditioned media express proangiogenic potential. Angiogenesis *in vitro* tests (Matrigel culture tubule formation and tubule formation capacity by spheroids formed by ASCs) suggest higher angiogenic potential of ASCs from young versus old donors and from long-term versus short-term cultures, but the differences are barely statistically significant and both young donor-derived and old donor-derived ASCs express proangiogenic capacity.

### 3.5. Endogenous *β*-Galactosidase Activity in ASC In Vitro Cultures

**Figure 5:**
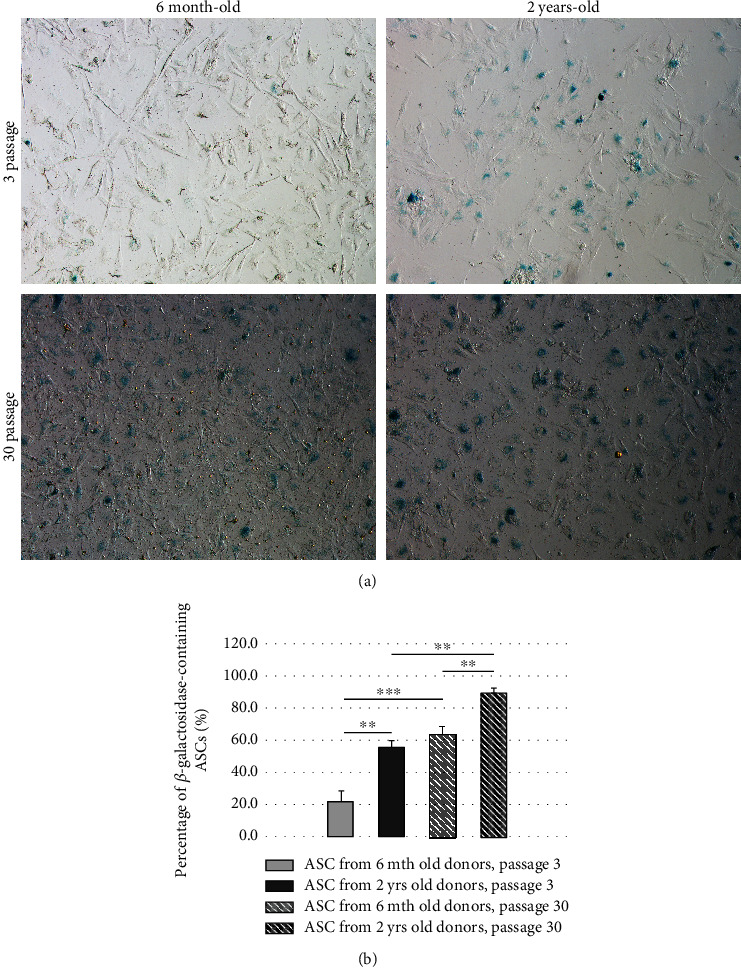
(a) Endogenous–*β*-galactosidase (blue) in ASC cultures from 6 mth donors, p. 3; 2 yrs. donors, p. 3; 6 mth donors, p. 30; 2 yrs. donors, p. 30. Light microscope, mag. 10x; (b) comparison of *β*-galactosidase-containing ASCs from 6 mth donors, p. 3; 2 yrs. donors, p. 3; 6 mth donors, p. 30; 2 yrs. donors, p. 30. Analyses were performed in duplicate ($M\;\underset{¯}{\pm }\text{ SE}$; *n* = 6; ^∗∗^*p* < 0.01; *p* < 0.001).

Highest SA-*β*-Gal levels were observed in ASCs from long-term cultures from old donors (Figure 5(a)). Levels of SA-*β*-Gal of old donors were higher when compared to their young counterparts and higher in ASCs from long-term cultures than short-term cultures. Statistical analysis of results (Figure 5(b)) allows to conclude at high levels (*p* < 0.01-*p* < 0.001) of statistical significance that the differences between SA-*β*-Gal levels in ASC cultures of young and old donors are smaller than the differences in SA-*β*-Gal levels in ASCs' short-term and long-term cultures, and the phenomenon is not depending on the cell donor age.

### 3.6. Result Summary

Summarized results are presented in Table 2. Differences in the analyzed ASC parameters are divided into the differences between ASCs derived from the young of old donors (6 mth or 2 yrs. old rats) and the differences between ASCs cultured for the short (3 passages) or long (30 passages) time *in vitro.*

## 4. Discussion

ASC belongs to the class of progenitor cells [10, 11] and does not have the “stemness” capacity, as the consequence it ages similarly (although slower) than the other adult somatic cells. There are some data suggesting the presence of the “anti-aging” mechanisms, like the activity of telomerase, but most published data documents the age-related changes (limited lifespan, telomere shortening, etc.) of the ASC population. Our experiments confirmed the aging and mortality of ASC population; on the other hand, we observed longer than average somatic cell *in vitro* proliferative potential of ASCs (over 30 *in vitro* passages, up to 6 months *in vitro* cell culture duration) and some intriguing phenomena like the increase of cell clonality (CFU-F percentage) along the cell culture duration. Our results confirm the findings of other authors that ASCs are cells in between typical adult somatic cell population and stem cells. Wistar Albino Galaxo strain is indeed frequently used as a model for epilepsy research. However, as we published before, no differences were detected between adipose-derived stem cells (ASCs) isolated from Wistar Albino Galaxo and Lewis rat tissue [12]. Quantity, phenotype, clonogenic, and differentiation potential of cells isolated from different localizations of adipose tissue from WAG and LEW/W rat strains were compared.

We have analyzed these cellular parameters, which are commonly recognized as important for the use of ASCs for therapeutic purposes in regenerative medicine. ASCs are widely used for the clinical experiments (not routine clinical applications in US and Europe) for the tissue regeneration purposes, implant construction, local stimulation of tissue healing, or the modulation of the host immune system activity [13–26]. Despite of many *in vitro* or *in vivo* experiments, there is still lack of consensus concerning the age limit of the donors of ASCs for clinical applications. The situation is similar as the beginnings of bone marrow transplantations, when both the age of bone marrow donor and the age of marrow recipients were initially set at the safe low level and stepwise increased along with gaining theoretical and clinical experience.

For the purpose of the study, we have selected several cell parameters, which are important for the attempted role of ASC in tissue repair. These parameters are also most commonly considered in the theoretical and translational studies on ASC role in tissue microenvironment regulation and tissue repair.

It is the common knowledge, confirmed by multiple experimental data, that somatic cell populations are aging in parallel with the lifespan of the host, and first of two aims of our study was to evaluate the age-related differences between ASCs derived from the 6 mth old rats (approximate equivalent of human age of 25 yrs.) and 2 yrs. old rats (corresponding to human age of 80 yrs.).

Our second purpose of the study was to evaluate the impact of the ASC *in vitro* cell culture duration on cell aging. The mechanism of cell aging resulting from multiple cell divisions is commonly known, one of the most important being the shortening of chromosome telomeres up to the level which arrest further cell divisions (the Hayflick rule). ASCs (along with the other members of the MSC family) are rather slowly dividing cells *in vivo*, and their increased activity is stimulated by the local tissue injury and repair processes [27, 28]. *In vitro* ASCs are frequently “expanded” in bioreactors, and the technique enabling to obtain higher cell numbers for clinical purposes and the published data on the impact of the culture duration on the quality of bioreactor-expanded cells are rather scarce and focused mostly on the analysis of possibly chromosomal abnormalities or possible cancerogenic transformation. Our data show that ASC aging caused by prolonged *in vitro* culture may be as important for their clinical applications, as the advanced age of the cell donor.

Like other authors, we did not observe any significant differences in the expression of ASC surface markers CD29 and CD90 depending both on donor animal age or *in vitro* cell culture duration [11, 29]. Both markers are used for classification of cells into MSC category, and their constant presence in all experimental groups confirms that ASCs, nevertheless of the animal age or *in vitro* culture duration, maintain their morphology and may be identified or FACS sorted on the basis of their surface markers. FACS analysis revealed that ASCs from aged individuals contain more granular structures than their counterparts from young donors—phenomenon nonexisting in “culture-old” versus “culture-young” cells. More granularity in ASC is probably associated with a greater mass of lysosomes in aged cells [30]. *β*-Galactosidase activity levels also correlate with the increase in lysosome mass (SA-*β*-Gal). Cell doubling time (growth rate) did not differ depending on cell donor age or cell culture duration. ASC metabolic activity, measured by MTS test, did not differ in ASCs derived from young and old animals. Similarly, there was no difference in ASC clonogenic potential (CFU-F test) of ASCs from young and old donors. Surprisingly, the metabolic activity and the percentage of colony-forming cells were significantly increased after long-term *in vitro* culture. The intriguing observation, to our knowledge not described yet by the other authors, may suggest that long-term *in vitro* culture allows to select less differentiated ASC subpopulation, enriched in clonogenic cells with higher metabolic activity. Such mechanism was observed in the other stem/progenitor cell populations stimulated for self-renewal activity, for example, during hematopoietic system regeneration after sublethal host irradiation or bone marrow transplantation. Age-related increase of the adipogenic and chondrogenic ASC potential *in vivo* and parallel decrease of osteogenic potential during aging of individuals were observed by the others, and we succeeded to confirm similar regularities for adipo- and chondrogenesis in *in vitro* model of cell aging dependent on cell culture duration [31–37]. Real-time PCR did not give consistent statistically significant results for adipogenesis-related marker genes.

ASCs are actively forming the elements of new blood vessels *in vitro* and are capable to stimulate neoangiogenesis *in vivo.* Several tests for angiogenic potential of ASC confirmed higher proangiogenic potential of ASCs from young rats when compared to ASCs from old animals. In contrary, 2 of 3 tests for angiogenic potential of ASCs *in vitro* (tubule formation potential and single spheroid summarized tubule length) have shown higher proangiogenic activity of cells from long-term cultures than of their short-term culture-derived counterparts. Another parameter (mean tubule length) in opposite suggested stronger proangiogenic potential of short-term culture-derived cells, and the diversity between tests does not allow for definite conclusions concerning changes of angiogenic potential related to the *in vitro* culture duration [4, 38–41].

*β*-Galactosidase level is commonly accepted marker of cell senescence. In our experiments, we have observed results consistent with the observations of other authors and “common logics,” where SA-*β*-Gal levels were higher in ASCs from senile donors than their young counterparts and higher at the end of the long-term *in vitro* culture than in early cell passages. The interesting and not yet published observation was the comparison of the ASC aging during the host lifespan (3 mth versus 2 yrs. old rats) with the aging of ASCs cultured for 30 passages (for 6 months) *in vitro*. The increases of SA-*β*-Gal levels were more intense during *in vitro* cell aging than during almost full lifespan of donor animal. Such observation is also the argument for need of careful and controlled use of bioreactors for the expansion of ASCs for clinical applications, since the extensive prolongation of the cell culture may result in obtaining cells more aged than their counterparts obtained from senile donors.

## 5. Conclusions

It may be concluded that the donor age does not affect phenotypical nor functional characteristics of mesenchymal stem cells to the extent which would affect their usefulness for the regenerative medicine applications. *In vitro* aging processes of MSCs resulting from long-term culture (e.g., bioreactor expansion for clinical purposes) may be equal or more intense than MSC aging resulting from advanced age of cell donors.

## Figures and Tables

**Table 1:** Comparison of the growth rate and doubling time of ASCs from passage 3 (p. 3) and passage 30 (p. 30) from 6 mth and 2 yrs. donor rats (Doubling Time Online Calculator).

ASC donor (rat) age and passage (p.) No.	Growth rate	Doubling time (h)
6 mth, p. 3	0.04 ± 0	18.90 ± 1.35
2 yrs., p. 3	0.04 ± 0	18.77 ± 0.43
6 mth, p. 30	0.04 ± 0	18.89 ± 1.72
2 yrs., p. 30	0.04 ± 0	18.13 ± 0.41

**Table 2:** Comparison of the ASC parameters analyzed in young versus old cell donors and in cells derived from short-term versus long-term cultures of ASCs. Descriptions: -: parameter similar in both groups, ↑: parameter higher, or ↓: lower than in corresponding group. ACAN and COMP—chondrogenesis marker genes, analyzed by real-time PCR. All the differences are significant at least at *p* < 0.05 (detailed data in text).

Parameter	Cell donor age		Time in culture	
	Young	Old	Short	Long
Surface markers CD29, CD90	-	-	-	-
Cell granularity	↓	↑	-	-
Growth rate (doubling time)	-	-	-	-
Metabolic activity	-	-	↓	↑
Clonogenic potential (CFU-F number)	-	-	↓	↑
Adipogenic potential	↓	↑	↓	↑
Chondrogenic potential	↓ (ACAN)	↑ (ACAN)	↓ (COMP)	↑ (COMP)
Osteogenic potential	↑	↓	-	-
Angiogenesis (tubule formation potential)	↑	↓	↓	↑
Angiogenesis (single spheroid summarized tubule length)	↑	↓	↓	↑
Angiogenesis (mean tubule length)	↑	↓	↑	↓
SA-*β*-Gal level	↓	↑	↓	↑↑

## Data Availability

Data are available on request from the authors.
